# Differences in anxiety, worry, and perceived stress among naturally cycling women and oral contraceptives users: a cross-sectional study investigating the role of contraceptive types

**DOI:** 10.1007/s00737-023-01405-1

**Published:** 2023-11-29

**Authors:** Melanie Kowalczyk, Monika Kornacka, Zofia Kostrzewa, Izabela Krejtz

**Affiliations:** 1 SWPS University, Institute of Psychology, Warsaw, Poland; 2 SWPS University, Institute of Psychology, Katowice, Poland

**Keywords:** Anxiety, Worry, Stress, Oral contraceptives, Menstrual cycle

## Abstract

The goal of our study was to test whether the types of OC affect the link between anxiety and its main maintenance factors: worry and perceived stress. Women are particularly at risk of being affected by excessive worrying, a core component of generalized anxiety disorder (GAD), and they are twice as likely as men to suffer from GAD. The literature suggests that gonadal hormones and types of oral contraceptives (OC) should be taken into account when exploring anxiety disorders in women, but the precise mechanism of this link remains understudied. We performed an observational cross-sectional study on a sample of 908 women, including 499 women naturally cycling (NC) and 409 taking OC (277 in the anti-androgenic group, 132 in the androgenic group). The participants filled in a battery of online questionnaires. Anxiety positively correlated with worry and perceived stress in the whole sample and in the three groups: androgenic OC, anti-androgenic OC, and NC. There was no significant difference between the groups on all the variables apart from the age of the participants. However, we found that women taking anti-androgenic OC had significantly higher levels of worry than NC women (after controlling for stress and age). The differences in OC types should be taken into account in future studies which might also lead to a better choice of OC based on women’s individual needs.

## Introduction

Generalized anxiety disorder has a prevalence of 4.5% worldwide (Stein et al. 2021). According to the Diagnostic and Statistical Manual of Mental Disorders (5th ed.; DSM–5; American Psychiatric Association 2013), GAD is manifested by excessive worry and anxiety about any given topic, a difficulty to control those worries and physical symptoms such as constant tiredness. GAD is related with higher levels of worry than any other anxiety disorder or psychiatric disorder (Olatunji et al. 2010). According to Borkovec et al. (1983), worry is defined as “a chain of thoughts and images, negatively affect laden and relatively uncontrollable” (p. 10). However, in the cognitive model of GAD (Wells 1999), worry can be seen as a positive coping strategy as the person believes that worrying serves a protective function to anticipate potential future threats. These beliefs reduce distress in the short-term but do not solve it, and the repetition of this strategy only reinforces distress in the long run (Andrews et al. 2010). Women are particularly susceptible to being affected by excessive worrying (Lal et al. 2014), they are twice as likely as men to suffer from GAD (Howell et al. 2001), and they are more likely to develop comorbid disorders (Howell et al. 2001; McLean et al. 2011).

One of the factors affecting both anxiety levels and physical symptoms might be naturally occurring hormonal fluctuations since women’s levels of estradiol and progesterone vary through the course of the menstrual cycle. Those hormonal fluctuations happen every month from women’s teenage years to middle age, and their impact should be considered to understand the onset and maintenance of anxiety disorders. Moreover, women in their reproductive years are susceptible to using hormonal contraceptives which also might have an impact on their anxiety levels (Beltz 2022; Laird et al. 2019). To understand the link between hormones and anxiety, it is also necessary to take into account the main trait dispositions involved, according to the literature, in the maintenance of anxiety: worry, and perceived stress, which positively correlates with levels of anxiety (Mirón et al. 2019). According to the preponderant models of GAD, the cognitive-behavioral model (Dugas et al. 2005), the cognitive model (Wells 1999), and the metacognitive model of GAD (Wells 2005), pathological worry can cause GAD (Behar et al. 2009). Additionally, worry can also enhance and maintain the physiological and cognitive activation caused by stress, resulting in higher and perseverative anxiety (Brosschot et al. 2006). The aim of the present study was to test whether the types of OC affect the link between anxiety and its main maintenance factors: worry and perceived stress.

### The menstrual cycle and the use of oral contraceptives

The menstrual cycle of women who cycle naturally usually lasts between 21 and 35 days and is divided into two parts: a follicular phase and a luteal phase (Fehring et al. 2006). The menstrual cycle starts with the first day of menstruation when both estradiol and progesterone levels are low. Menstruation is part of the follicular phase, which is characterized by low levels of progesterone and by levels of estradiol that continuously increase until reaching a peak level before ovulation. The luteal phase starts after ovulation and shows increasing levels of progesterone and a slight increase in estradiol, followed by a decrease of both hormones towards the end of the menstrual cycle (Abraham et al. 1972).

The use of OC is linked with a noticeable decrease in levels of estradiol and progesterone (Hampson 2020). During the periods of active pill use, the levels of these two hormones are similar to the levels of NC women at the beginning of their menstrual cycle (Elliott-Sale et al. 2013). Moreover, levels of testosterone, a form of androgen, are down by 50 to 60% in women taking OC compared to NC women (Hampson 2020). Combined OC usually contain ethinylestradiol and progestins, which can be androgenic or anti-androgenic. Androgenic progestins are derived from testosterone, while anti-androgenic progestins block androgen receptors (Raudrant and Rabe 2003). The androgenic OC slightly counteract the effects induced by the decreasing levels of testosterone in OC users, but the anti-androgenic OC reinforce them (Zimmerman et al. 2014). Androgenic and anti-androgenic progestins show differences in the brain structures of women taking them compared to NC women (Pletzer et al. 2015). For instance, androgenic progestins masculinize the brain activation patterns in spatial skills (Beltz 2022). Anti-androgenic progestins are often used, not only as a form of contraception, but also to stop androgenization symptoms such as acne, hirsutism, or alopecia (Schindler 2013).

Considering the differential impact of the various types of OC on womens’ health, it seems crucial to take this distinction into account while studying women taking OC. First, because of the scale of the phenomenon, based on the data gathered by the United Nations (2019), 16% of women of reproductive age use OC worldwide, which make them the fourth most common contraceptive method, with the highest prevalence in Europe (17.8%). Second, the differential impact of the various types of OC might potentially explain the contradictory results presented in the literature linking anxiety to OC.

### The link between hormones and anxiety

The literature is consistent on the fact that anxiety symptoms are related to women’s levels of estrogen (Borrow and Handa 2017) and progesterone (Reynolds et al. 2018); however, this relation and its precise mechanism are still understudied. A literature review by Robakis et al. (2019) on the link between OC and mood concluded that OC use does not impact women’s mood in a negative manner in the general population. A meta-analysis conducted by Motter (2019) in her thesis showed that OC use can sometimes even enhance mood. However, the literature review conducted by Laird et al. (2019) on the link between OC, mental disorders, and cognitive functions described a lack of consistency in the presented results. This lack of consistency might be due to the following limitations: a lack of distinction between different kinds of OC and a lack of studies on women suffering from anxiety symptoms. According to a review by Beltz (2022), most studies conducted on hormonal contraception also suffer from small sample sizes. This review underlined as well that women taking hormonal contraceptives are similar to women having experienced chronic stress in that they report the same levels of anxiety and their cortisol levels are similar, while they show a blunted acute stress response.

### Objective

Taking into account the gap in the literature, i.e., the lack of comparison between the different kinds of OC, the aim of the present study was to test whether the types of OC affect the link between anxiety and its main maintenance factors: worry and perceived stress. In order to do so, we conducted an online cross-sectional study with a large sample. Our hypotheses were that (1) anxiety, worry, and stress are linked together; (2) the levels of anxiety differ between NC women and women taking OC, depending on the types of OC they are using; (3) there is a difference in the levels of worry and perceived stress between NC women and women taking OC, depending on the types of OC they are using.

## Methods

### Participants

In total, 1289 women participated in the study. 908 of them were included with 499 cycling naturally and 409 taking OC. The OC users were further classified according to the androgenicity of their OC (Mathur et al. 2008; Raudrant and Rabe 2003): 277 participants for the anti-androgenic group and 132 for the androgenic group. The inclusion criteria for participants in the study were as follows: being between 18 and 45 years of age, speaking Polish, taking OC or being NC. The exclusion criteria were as follows: using other forms of hormonal contraception (Pahnke et al. 2019), using a copper intrauterine device, having undergone sterilization, having stopped taking OC in the past 6 months (Hidalgo-Lopez and Pletzer 2017), being pregnant or breastfeeding (Welz et al. 2016). We did not consider the phases of the menstrual cycle or the phases of the OC (active/inactive). In order to determine the sample size for a fixed effects ANOVA, a power analysis was conducted using G*Power (Faul et al. 2007). With a medium effect size of .25, the results of the power analysis showed that a minimum of 252 participants (84 participants per group) would be needed to achieve an appropriate power level for this study.

### Materials

We used the GAD-7 Scale (Spitzer et al. 2006), the Penn State Worry Questionnaire (PSWQ; Meyer et al. 1990), and the Perceived Stress Scale (PSS; Cohen et al. 1983). The GAD-7 is a 7-item scale used to screen for GAD by measuring levels of anxiety. The participants reply to the question “Over the last 2 weeks, how often have you been bothered by the following problems?” regarding different symptoms related to GAD such as “Feeling nervous, anxious, or on edge” and using a scale from 0 (“not at all”) to 3 (“nearly every day”). The Cronbach alpha in our study was *α* = .91, which indicated a high internal consistency.

The PSWQ is composed of 16 items measuring trait levels of worry. The instructions for this questionnaire are as follows: “Rate each of the following statements on a scale of 1 (“not at all typical of me”) to 5 (“very typical of me”). An example of an item from the scale is as follows: “Many situations make me worry.” The Cronbach alpha was high (*α* = .91).

The PSS is a 10-item questionnaire and is the most widely used psychological instrument for measuring the perception of stress. The instructions indicate that the questions are referring to the participants’ feelings and thoughts in the last month and that the participants have to express how often they felt or thought a certain way on a scale from 0 (“never”) to 4 (“very often”). The Cronbach alpha was high (*α* = .89).

### Procedure

The participants were recruited via an advertisement posted on social media, which invited them to join a scientific study aiming at examining the link between the menstrual cycle and anxiety in women who take OC and women who cycle naturally. The advertisement presented a description of the study and the necessary criteria to take part in it. The participants who accepted to take part in the study provided informed consent and filled in a battery of questionnaires online. The data were collected between February and November 2022. All the questionnaires were presented in their Polish version. The participants did not receive any compensation for taking part in the study. The study was conducted in compliance with the Helsinki Declaration, and the research protocol was approved by the Ethics Committee of our institution. This study is part of a larger study whose results will be published separately.

## Results

Our analyses took on the following structure: first, a one-way ANOVA with group (NC, androgenic-OC, anti-OC) as a between-subjects factor was run for age. There was a significant difference in age between the groups, *F*(2,905) = 19.47, *p* < .001. Post hoc comparisons showed that women taking anti-androgenic OC were significantly younger than the NC women and the women taking androgenic OC. Table 2 presents the descriptive statistics for these comparisons. We then conducted a Pearson’s correlation test to examine the link between the variables. Afterwards, we conducted an ANCOVA in order to check whether there was a difference between groups related to the levels of worry with age and anxiety as covariates and then an ANCOVA with stress and age as covariates. The post hoc comparisons for the ANCOVA were adjusted with the Bonferroni correction. We used bootstrapping with 1000 samples to estimate the post hoc comparisons.

### Relationship between anxiety, worry, and stress

We first conducted an examination of the correlations between the variables. As expected, anxiety correlated positively with worry and perceived stress in the general sample and in the three groups: androgenic OC, anti-androgenic OC, and NC. The correlation coefficient values were transformed into z scores and compared for significance. There were no significant differences in correlation coefficients between groups.

Age correlated negatively with anxiety, worry, and stress in the general sample and in the androgenic OC group. In the anti-androgenic OC group and in the NC group, only worry correlated negatively with age. There was a significant difference in correlation coefficients between the androgenic OC group and the anti-androgenic OC group (*z* = −2.12, *p* = .03), and between the androgenic OC group and the NC group (*z* = −2.60, *p* = .009): the correlation was the highest in the androgenic group. The correlations are shown in Table 1.

**Table 1:** Pearson’s correlation coefficients

	General sample (*n* = 908)	Androgenic OC (*n* = 132)	Anti-androgenic OC (*n* = 277)	NC (*n* = 499)
Anxiety				
Worry	.58**	.65**	.55**	.59**
Perceived stress	.68**	.69**	.69**	.67**
Age	−.10**	−.34**	−0.03	−0.08
Worry				
Perceived stress	.70**	.71**	.71**	.69**
Age	−.21**	−.40**	−.19**	−.16**
Perceived stress				
Age	−.09**	−.34**	−0.03	−0.05

Correlation is significant with **p < .01

### Differences in levels of anxiety, worry, and stress between groups

In order to check whether there was a difference between groups related to the levels of anxiety, worry, and stress of the participants, we conducted an ANCOVA separately for each variable, and age was added as a covariate in each of the analyses. The analysis did not support any differences between groups in anxiety levels, *F*(2, 904) = 1.81, *p* = .165, worry, *F*(2, 904) = 1.15, *p* = .318, or perceived stress, *F*(2, 904) = 1,58, *p* = .207. Table 2 presents the descriptive statistics for these comparisons.

**Table 2:** Descriptive statistics

	General sample (*N* = 908)		Androgenic OC (*n* = 132)		Anti-androgenic OC (*n* = 277)		NC (*n* = 499)	
	*M*	*SD*	*M*	*SD*	*M*	*SD*	*M*	*SD*
Age	28.54	6.66	28.89	6.84	26.52	5.92	29.56	6.77
Anxiety	7.06	4.69	6.42	4.61	7.09	4.63	7.21	4.74
Worry	55.06	13.96	53.39	13.97	56.62	13.75	54.64	14.01
Perceived stress	21.47	7.12	20.87	7.84	21.26	7.16	21.75	6.89

### Differences in worry levels between groups with anxiety, stress, and age as covariates

In order to check whether there was a difference in worry levels between groups related to the levels of anxiety and stress of the participants, we conducted an ANCOVA with anxiety, stress, and age as covariates. After controlling for the levels of anxiety and age, the difference in the levels of worry between groups was not significant, *F*(2, 903) = 1.18, *p* = .307. However, after controlling for the levels of stress and age, a significant difference in levels of worry between groups emerged, *F*(2, 903) = 3.12, *p* = .045. Post hoc analysis was conducted using the bootstrapping procedure in order to obtain more accurate results. The post hoc analysis with 1000 bootstrapped samples indicated that women taking anti-androgenic OC had significantly higher levels of worry (*M* = 56.62, *SD* = 13.75) than NC women (*M* = 54.64, *SD* = 14.01) (*p* = .023).

## Discussion

In this large cross-sectional study, we examined whether women taking different types of OC and NC women differ in their levels of anxiety and its main maintenance factors: worry and perceived stress. We found no differences between groups on all the measures. We did find a significant difference between the groups in age, with the anti-androgenic OC group being younger than the androgenic OC group and the NC group. After controlling for age and levels of stress, we discovered that women taking anti-androgenic OC had higher levels of worry than women NC women. Nevertheless, a conservative conclusion should be made until the finding is replicated in another independent sample.

According to the literature, hormonal contraceptives in general moderate the association between worry and an enlarged error-related negativity (Louis et al. 2022). The error-related negativity is an electrophysiological marker appearing in the brain after an error in a cognitive task and is strongly related to anxiety, especially in women. Still, according to Louis et al. (2022), the association between worry and the enlarged error-related negativity was strong and only existed in hormonal contraceptive users but not in NC women. Our findings are consistent with Louis et al. (2022) in the sense that hormonal contraception is associated with levels of worry. We know from the literature that women are more likely to be affected by excessive worry than men (Lal et al. 2014). This might explain why anti-androgenic OC, which reduce the levels of male hormones, are linked with higher levels of worry. However, the latest literature review by Hampson (2023) underlines that the association between ethinylestradiol and certain progestins might also have an impact on neurotransmitters such as serotonin or dopamine in rats, which could also explain the difference between the levels of worry in androgenic and anti-androgenic OC users.

### Future studies

Our study followed the recommendations of previous literature reviews which underlined the necessity for large sample sizes in studies related to the link between hormonal contraception and anxiety (Beltz 2022), and the need to analyze the results according to the different types of OC (Laird et al. 2019). Both literature reviews noted that the results of previous studies were inconsistent which might have been due to the fact that the different types of OC were not taken into account. In our study, we can see that there is a significant difference in levels of worry between the groups taking anti-androgenic and androgenic OC. Previous studies might have missed these differences in their results if they grouped all the OC users together. Future studies on the link between anxiety and OC should take into account the differences between OC types in order to provide the most accurate results. The review by Hampson (2023) also underlines the need to compare OC types based on their ethinylestradiol levels and to update older findings about the impact of the association between ethinylestradiol and different types of progestins on neurotransmitters.

Moreover, the literature review by Robakis et al. (2019) and the meta-analysis by Motter (2019) showed that OC use is not linked with a decrease in mood in healthy women but the literature reviews by Laird et al. (2019) and Beltz (2022) concluded that the results are not consistent for women suffering from an anxiety disorder. Future studies should be conducted on women having obtained an actual diagnosis for an anxiety disorder in order to be able to compare them to healthy women. If a link was found between the use of OC, especially anti-androgenic OC, and anxiety disorders, then the prescription of OC should be made according to the individual needs of women and taking into account their risk of developing GAD.

Welz et al. (2016) underlined that studies on the menstrual cycle are often performed with a cross-sectional design and would benefit from using ambulatory assessments to better understand the differences between the phases of the menstrual cycle and the daily changes in women’s affect. Indeed, an intensive repeated-measures design would allow a more detailed analysis of the differences between the groups and to control for the different phases of the menstrual cycle in NC women in their daily functioning. Welz et al. (2016) used ecological momentary assessment to perform this kind of study, and more studies with this methodology are warranted.

The review by Laird et al. (2019) underlined the need to conduct more studies that would check the actual levels of hormones at least once in each phase, both in OC users (active/inactive pill phase) and in NC women (menstrual cycle phases), in order to obtain the most accurate results and comparisons between both groups. However, the most accurate results could be obtained when testing the OC users before and after they have started taking OC in order to account for changes directly induced by the treatment, such as in Gingnell et al. (2016).

The limitations of our study were the cross-sectional design which does not allow us to draw any causal conclusions, the use of self-report measures which might not always offer the best accuracy, and the recruitment method (social media) which is linked with a selection bias and does not allow to generalize the results to the whole population. Other limitations present in our study were the lack of the following: participants who had an actual anxiety disorder diagnosis, testing for hormonal levels, verifying whether the participants were taking the OC for contraceptive or gynecological reasons, comparing the different dosages of ethinylestradiol in the OC and analyzing the results depending on the menstrual cycle phase. Our research project includes future studies that will take into account those variables in a daily diary study and a laboratory study.

## Conclusion

To conclude, the main contribution of the present large-sample correlational study on women taking OC and NC is twofold. First, we demonstrated that there is a link between oral contraception and worry levels. Second, by differentiating between OC types, and controlling for anxiety and stress perception, we observed that women taking anti-androgenic OC presented higher levels of worry than NC women. As a result, we suggest that controlling for OC type is a significant factor potentially responsible for the observed inconsistencies in the literature. We also underline that worry is an important risk factor for developing anxiety, and it should be monitored, particularly in women taking anti-androgenic OC.

## Data Availability

The data that support the findings of this study are available from the corresponding author, upon request.
